# PD-L1 expression correlation with metabolic parameters of FDG PET/CT and clinicopathological characteristics in non-small cell lung cancer

**DOI:** 10.1186/s13550-020-00639-9

**Published:** 2020-05-19

**Authors:** Xiaodong Wu, Yan Huang, Qingping Zhao, Lei Wang, Xiao Song, Yi Li, Lei Jiang

**Affiliations:** 1 Department of Nuclear Medicine, Shanghai Pulmonary Hospital, Tongji University School of Medicine, 507 Zhengmin Road, Shanghai, 200433 China; 2 Medical College of Soochow University, Suzhou, 215123 China; 3 Department of Pathology, Shanghai Pulmonary Hospital, Tongji University School of Medicine, 507 Zhengmin Road, Shanghai, 200433 China; 4 Department of Oncology, Shanghai Pulmonary Hospital, Tongji University School of Medicine, 507 Zhengmin Road, Shanghai, 200433 China; 5 Department of Thoracic Surgery, Shanghai Pulmonary Hospital, Tongji University School of Medicine, 507 Zhengmin Road, Shanghai, 200433 China

**Keywords:** FDG, PET/CT, PD-L1, Non-small cell lung cancer

## Abstract

**Background:**

Immunotherapy targeting programmed cell death 1 (PD-1) or its ligand 1 (PD-L1) has shown promising results in non-small cell lung cancer (NSCLC) patients. Exploring PD-L1 expression could help to select NSCLC candidates for immunotherapy. Fluorine-18 fluorodeoxyglucose (FDG) PET/CT could provide phenotypic information on malignant tumors. Thus, this study investigated PD-L1 expression correlation with metabolic parameters of FDG PET/CT and clinicopathological characteristics in NSCLC.

**Methods:**

FDG PET/CT metabolic parameters including maximum standard uptake (SUVmax), metabolic tumor volume and total lesion glycolysis of primary lesion (MTV-P, TLG-P), and combination of primary lesion and metastases (MTV-C, TLG-C) were compared with PD-L1-positive expression in patients with NSCLC. Moreover, clinicopathological characteristics, including age, gender, smoking history, serum tumor markers, tumor location, size, TNM stage, and genetic mutation were also reviewed.

**Results:**

All 374 patients (215 men; 159 women; age 63 ± 9 years) included 283 adenocarcinomas (ACs) and 91 squamous cell carcinomas (SCCs). PD-L1 expression was positive in 27.8% (104/374) cases. SUVmax, TLG-P, and TLG-C of PD-L1 positivity were significantly higher than PD-L1 negativity. Moreover, PD-L1 expression was obviously correlated with man, smoking, and central NSCLC. If ACs and SCCs were separately analyzed, PD-L1 positivity in ACs and SCCs was 21.6% (61/283) and 47.5% (43/91), respectively, and only SUVmax was obviously associated with PD-L1 expression. Furthermore, multivariate analysis revealed that only SUVmax was an independent predictor of PD-L1 positive expression in overall NSCLC, AC, and SCC. Using a SUVmax cut-off value of 12.5, PD-L1 status of NSCLC was predicted by FDG PET/CT with sensitivity, specificity, and accuracy of 65.4%, 86.7%, and 80.7%, respectively.

**Conclusions:**

PD-L1 expression of NSCLC was related to SUVmax, TLG, man, smoking, and central location. However, only SUVmax was an independent predictor of PD-L1 positivity, which could help to explore the existence of immune checkpoints.

## Background

Lung cancer is the most common cancer and the leading cause of cancer death all over the world [1], and non-small cell lung cancer (NSCLC) is the most commonly diagnosed histological subtype [2–4]. Despite the development in treatment for NSCLC such as thoracoscopic surgery, chemotherapy, radiotherapy, and targeted therapy [5, 6], the overall 5-year survival rate is still poor [7]. According to the data from 2009 to 2015, the 5-year survival rate of patients with lung cancer is as low as 19.4%, while that of patients with distant metastasis is only 5.2% [8]. Nowadays, many studies indicated that immunotherapy targeting programmed cell death ligand-1 (PD-L1) could provide a promising new way for the treatment of NSCLC [9, 10]. Antibodies blocking PD-1/PD-L1 pathway can enhance anti-tumor immunity to achieve the goal of killing cancer cells [11]. Thus, it is necessary to explore the existence of immune checkpoints and to select NSCLC candidates for immunotherapy.

At present, immunohistochemistry is the main method for identifying tumor PD-L1 expression, which requires surgical or biopsied tumor specimens from patients with NSCLC [12]. However, these procedures such as surgery, transbronchial lung biopsy, or CT-guided biopsy are invasive and even failed due to the patients’ physical condition or unqualified specimens. Fluorine-18 fluorodeoxyglucose (FDG) PET/CT is a noninvasive imaging modality and can be applied even if specimens from patients are not available, which has been widely applied to predict many molecular phenotypes of malignant tumors, such as histological types, tumor differentiation, proliferation, hypoxia, and genetic mutation [13–17].

The previous studies have confirmed that tumor expression of PD-L1 was related to high glucose metabolism of PET/CT in NSCLC [18–22]. However, almost all previous studies focused on the correlation of the FDG PET/CT metabolic parameter of maximum standard uptake (SUVmax) with PD-L1 expression [18–22]. SUVmax is easy to determine and reflects the maximum uptake of glucose by metabolically active lesions. Volume-based PET parameters such as metabolic tumor volume (MTV) and total lesion glycolysis (TLG) represent glucose activity in the entire tumor mass, which could also reflect the metabolic status of the malignant tumors [23]. However, at present, little is known about the association of PD-L1 with MTV and TLG.

Therefore, in this study, FDG PET/CT findings and metabolic parameters of SUVmax, MTV and TLG of primary lesion (MTV-P and TLG-P), and combination of primary lesion and metastases (MTV-C and TLG-C) were retrospectively analyzed in 374 patients with NSCLC. We investigated tumor PD-L1 positive expression correlation with these metabolic parameters and patients’ clinicopathological characteristics and analyzed their roles in predicting PD-L1 expression in NSCLC.

## Materials and methods

### Patients

From January 2017 to August 2018, 374 NSCLC patients with definite PD-L1 expression results who underwent FDG PET/CT examination in our department were included in this study according to the following criteria: (1) the interval between PET/CT examination and pathological diagnosis was no more than 2 weeks; (2) only adenocarcinoma (AC) and squamous cell carcinoma (SCC) were included, and other NSCLC subtypes were excluded; (3) there was no other cancer history or coexisting other malignant tumors; and (4) the patients did not receive any prior systemic or local tumor therapy.

### FDG PET/CT scan

These scans were performed on a Biograph 64 system (Siemens Healthineers, Erlangen, Germany) with a 21.6 cm axial field of view. The patients were required to fast for at least 6 h prior to imaging, and serum glucose levels were kept lower than 7.4 mmol/l. Images were captured ~60 min after intravenous administration of 3.7 MBq of FDG per kilogram of body weight. Six or 7 bed positions from the base of the skull to the mid-thighs were imaged. PET images were acquired for 2.5 min per bed position. CT was performed on the same scanner without contrast administration, and CT scan data were collected under the following conditions: 120 kV, 101 mA (adjusted by auto mA), and a gantry rotation speed of 0.5 s. All the CT scans were conducted via 5-mm-thick axial slices. PET images were reconstructed at 200 × 200 pixels using a Gaussian filter of 5.0 mm full width at half maximum value. All image reconstructions were performed with the ordered-subset expectation-maximization algorithm, incorporating a CT-based transmission map.

Regions of interest (ROIs) were defined around the pulmonary lesions, and the maximum and mean values of an ROI were defined as the SUVmax and SUVmean, respectively. MTV-P and MTV-C were computed with 40% of SUVmax as a threshold. TLG-P and TLG-C were calculated according to the following formula: TLG = SUVmean × MTV [22, 24]. PET/CT imaging results were analyzed and interpreted by 2 experienced nuclear medicine physicians who were unaware of the patients’ clinical information, other conventional imaging findings, and pathology results.

### Histopathological and genetic analyses

Tumor specimens in this study were obtained after surgical excision or biopsy. The specimens were carefully examined, and the part with more malignant cells, less differentiated cells, and less hemorrhage and necrosis were subjected to histopathological quantification of PD-L1 expression. The platform of Dako Link 48 and the antibody of Dako 22C3 were used for PD-L1 staining. Tumor proportion score (TPS) was recorded as the percentage of PD-L1 positive tumor cells over all tumor cells, and TPS ≥ 1% were considered PD-L1 positive expression [22, 25].

Moreover, we recorded and counted gene mutations in patients who had undergone genetic testing, including epidermal growth factor receptor (*EGFR*), Kirsten rat sarcoma viral oncogene homolog gene (*KRAS*), echinoderm microtubule-associated protein-like 4-anaplastic lymphoma kinase fusion gene (*EML4-ALK*), v-Raf murine sarcoma viral oncogene homolog B1 (*BRAF*) proto-oncogene, and c-Ros oncogene 1 (*ROS1*). These histopathological and genetic results were reviewed by 2 experienced pathologists.

### Statistical analysis

Data are expressed as the mean ± SD. Univariate analysis of differences between groups was determined using the independent *t* test, one-way ANOVA, or chi-squared test, where applicable. Multivariate analysis of the relationship between PD-L1 expression and selected factors which have significance via univariate analysis was performed by logistic regression. The receiver operating curve was performed to determine the optimal SUVmax cut-off value using bootstrapping of R package for predicting the expression of PD-L1, and random sampling was conducted 1000 times from all 374 cases. A *P* value <0.05 was considered statistically significant. SPSS 21.0 software for Windows (IBM Corp., Armonk, NY, USA) was used for the statistical analysis.

## Results

### Patients’ clinicopathological characteristics

All 374 patients included 215 men and 159 women, with an average age of 63 ± 9 years (range 32–87 years). Smoking history was found in 205 patients and all of them were men. A total of 359 out of 374 patients were examined for serum tumor markers, and 73 had elevated levels of cytokeratin 211 (CYFRA211, reference range < 3.3 ng/ml), 40 had elevated carcinoembryonic antigen (CEA, <10 μg/l), 8 had elevated neuron-specific enolase (NSE, <20 ng/ml), and 1 had elevated pro-gastrin-releasing peptide (proGRP, <3 ng/ml), respectively. No patient was found to have elevated squamous cell carcinoma antigen (SCCA, <3 ng/ml).

All patients presented with solitary primary lesion with a mean diameter of 32 ± 15 mm (range 7–120 mm), including 283 ACs and 91 SCCs. Fourteen cases were central tumors and 360 were peripheral. The TNM stages of 374 cases were as follows: 235 cases were at stage I, 77 at stage II, 60 at stage III, and 2 at stage IV. PD-L1 positive expression was detected in 104 of 374 cases, and the positive rate was 27.8%. A total of 349 out of 374 patients underwent genetic testing, *EGFR* mutation was found in 145 patients, *KRAS* mutation in 26, *EML4-ALK* fusion in 5, and *ROS1* mutation in 2, respectively. No *BRAF* mutation was observed.

### PET/CT

FDG uptake was avid in all 374 primary lung tumors with the average SUVmax, MTV-P, and TLG-P of 9.5 ± 5.5 (1.1–24.5), 13.3 ± 19.1 (0.7–168.9), and 96.8 ± 192.2 (1.5–1457.1), respectively. Among 374 patients, 88 cases had local and distant metastases, including 86 with only lymph node metastases, 1 coexistence with lymph node and pleural metastases, and other one coexistence with lymph node and adrenal metastases. Therefore, the mean MTV-C and TLG-C of all cases were 14.1 ± 19.5 (0.7–168.9) and 99.3 ± 193.9 (1.5–1457.1), respectively.

### PD-L1 correlation with PET/CT and clinicopathological features in NSCLC

**Fig. 1:**
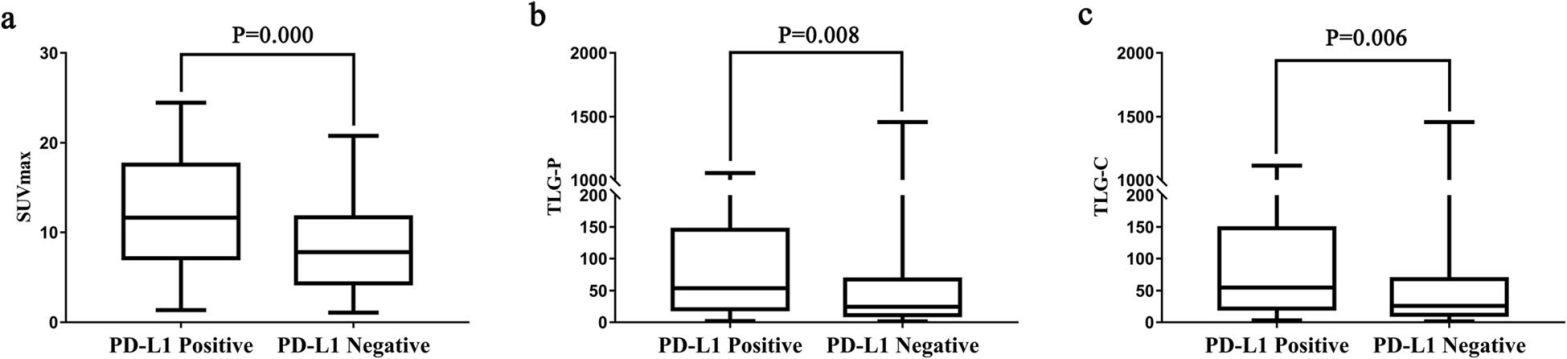
The correlation between PD-L1 expression and SUVmax (**a**), TLG-P (**b**), and TLG-C (**c**) in overall NSCLC

SUVmax, TLG-P, and TLG-C in PD-L1 positive tumors were significantly higher than those in PD-L1 negative tumors *(P* < 0.05) (Table 1, Fig. 1). And no significant differences in MTV-P and MTV-C were found between PD-L1 positive and negative groups (*P* > 0.05, Table 1). There was an obvious correlation between PD-L1 expression and man, smoking, and central NSCLC (*P* < 0.05, Table 1). No significant differences in other clinicopathological features such as age, serum tumor markers, tumor size, TNM stage, and genetic mutation were found between PD-L1 positive and negative groups (*P* > 0.05, Table 1).

**Table 1:** Univariate analysis of the relationship between PD-L1 expression and PET/CT metabolic parameters, and clinicopathological characteristics

	Overall (*n* = 374)			Adenocarcinoma (*n* = 283)			Squamous cell carcinoma (*n* = 91)		
Variable	PD-L1 positivity (*n* = 104)	PD-L1 negativity (*n* = 270)	*P* Value	PD-L1 positivity (*n =* 61)	PD-L1 negativity (*n* = 222)	*P* Value	PD-L1 positivity (*n* = 43)	PD-L1 negativity (*n* = 48)	*P* Value
**Sex,*n***									
Male	72	143	0.004*	30	96	0.409	42	47	>0.999
Female	32	127		31	126		1	1	
**Age (mean ± SD, range, years)**	62 ± 9 (36–87)	63 ± 8 (32–83)	0.317	60 ± 10 (36–87)	63 ± 9 (32–86)	0.056	65 ± 7 (46–80)	66 ± 8 (43–83)	0.684
**Smoking history,*n***									
Yes	71	134	0.001*	30	90	0.227	41	44	0.680
No	33	136		31	132		2	4	
**Location,*n***									
Central	9	5	0.004*	0	0	/	9	5	0.245
Peripheral	95	265		61	222		34	43	
**Diameter (mean ± SD, range, mm)**	33 ± 15 (7-80)	32 ± 16 (8-110)	0.303	30 ± 12 (15-70)	29 ± 12 (8-120)	0.344	38 ± 18 (7-80)	45 ± 23 (11-110)	0.090
**Cyfra211,*n***									
High level	27	46	0.051	5	24	0.545	22	22	0.659
Normal level	73	213		54	190		19	23	
**TNM stage,*n***									
I	57	178	0.139	35	159	0.136	22	19	0.314
II	28	49		11	31		17	18	
III	19	41		15	31		4	10	
IV	0	2		0	1		0	1	
**PET-CT parameters(mean ± SD,range)**									
SUVmax	12.4 ± 6.0 (1.4-24.5)	8.3 ± 4.9 (1.1-20.8)	0.000*	10.0 ± 5.0 (1.4-23.2)	7.3 ± 4.5 (1.1-20.7)	0.000*	16.0 ± 5.6 (3.9-24.5)	13.2 ± 3.7 (4.9-20.8)	0.036*
MTV-P	14.7 ± 18.3 (0.8-86.0)	12.7 ± 19.5 (0.7-168.9)	0.425	8.2 ± 6.8 (0.8-34.6)	9.6 ± 13.2 (0.7-139.8)	0.362	23.9 ± 24.7 (0.8-86.0)	27.4 ± 33.5 (0.8-168.9)	0.581
TLG-P	143.0 ± 234.2 (1.9-1055.6)	78.9 ± 170.2 (1.5-1457.1)	0.008*	58.3 ± 81.4 (1.9-547.7)	46.7 ± 92.5 (1.5-947.0)	0.512	263.0 ± 316.0 (4.0-1055.6)	233.4 ± 316.1 (3.0-1457.1)	0.722
MTV-C	15.8 ± 19.1 (0.8-86.0)	13.4 ± 19.7 (0.7-168.9)	0.322	9.2 ± 7.2 (1.0-34.6)	10.2 ± 13.4 (0.7-142.4)	0.523	25.2 ± 25.9 (0.8-86.0)	28.7 ± 33.4 (0.8-168.9)	0.603
TLG-C	147.3 ± 237.2 (3.0-1112.9)	80.7 ± 171.1 (1.5-1457.1)	0.006*	62.2 ± 83.5 (3.0-547.7)	48.3 ± 93.4 (1.5-958.3)	0.423	268.1 ± 320.3 (4.0-1112.9)	236.2 ± 317.2 (3.0-1457.1)	0.698
**EGFR mutation,*n***									
Positive	32	113	0.059	29	113	0.787	3	0	0.104
Negative	64	141		27	97		37	44	

*P Value <0.05

**Fig. 2:**
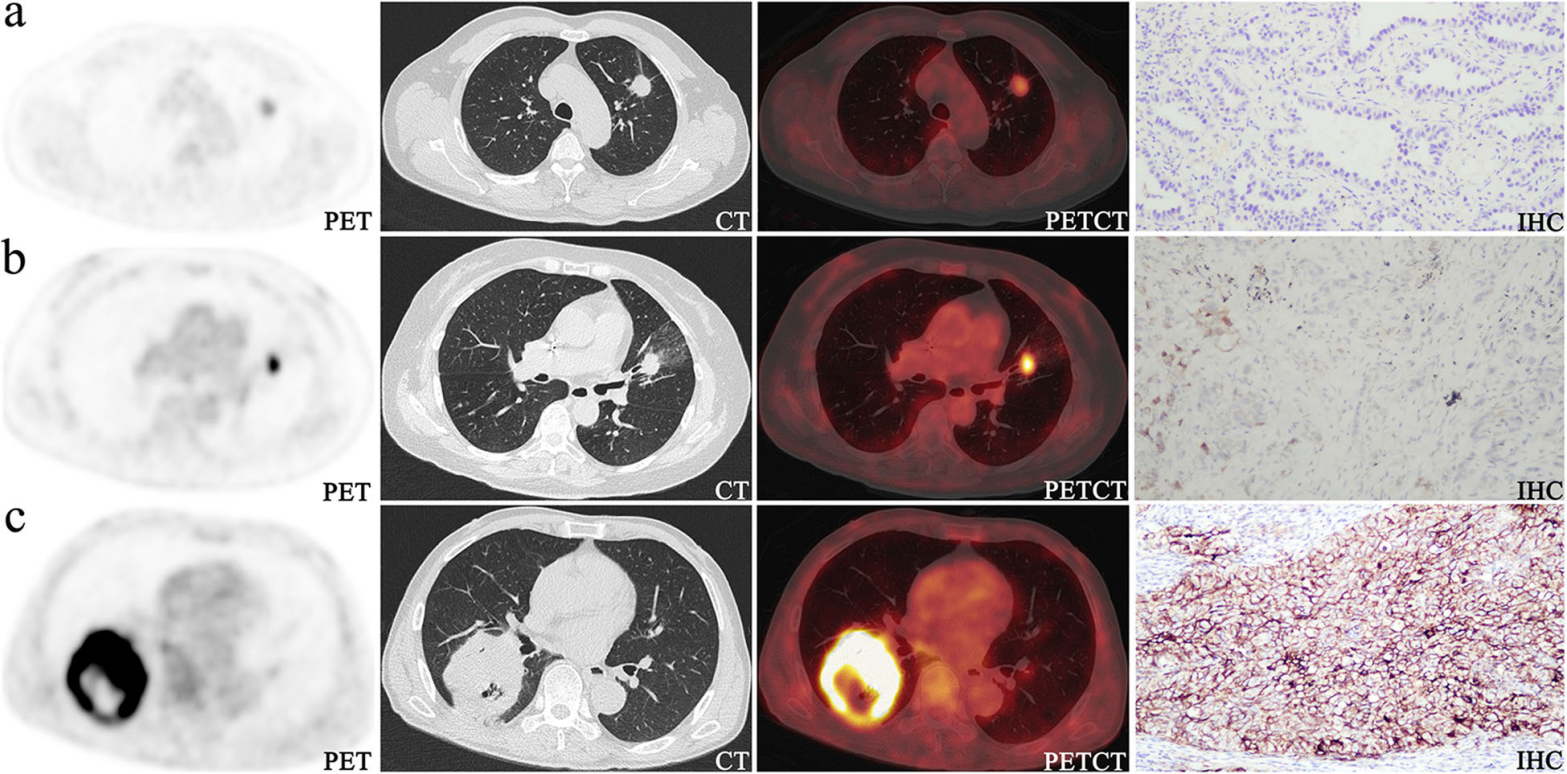
Representative PET, CT, fused, and immunohistochemistrical (IHC, ×100) pictures. **a** A 61-year-old man with left lung AC had an SUVmax of 4.9 and negative PD-L1 expression. **b** A 66-year-old woman with left lung AC had an SUVmax of 11.6 and positive PD-L1 expression during 1–49%. **c** A 69-year-old man with right lung SCC had an SUVmax of 21.3 and positive PD-L1 expression during 50–100%.

**Fig. 3:**
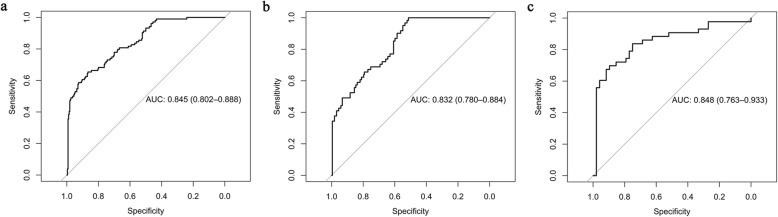
Bootstrapping analysis of the ability of SUVmax to predict PD-L1 expression in overall NSCLC (**a**), AC (**b**), and SCC (**c**)

Multivariate analysis showed that only SUVmax was an independent predictor of PD-L1 expression in NSCLC (Table 2, Fig. 2). Furthermore, the optimal SUVmax threshold to predict PD-L1 expression of NSCLC was investigated. The best SUVmax cut-off value was determined to be 12.5 using bootstrapping analysis and the area under the curve was 0.845 (95% CI 0.802–0.888). PD-L1 status of NSCLC could be predicted by SUVmax with sensitivity, specificity, and accuracy of 65.4%, 86.7%, and 80.7%, respectively (Fig. 3a).

**Table 2:** Multivariate analysis of the relationship between PD-L1 expression and selected factors which have significance via univariate analysis by logistic regression SUVmax as an independent predictor of PD-L1 expression by multivariate analysis

	Factor	Odds ratio	95% confidence interval	*P* value
**Overall (*n*= 374)**	Sex	0.38	0.07–2.11	0.268
	Smoking	3.86	0.70–21.18	0.121
	Location	2.55	0.757–8.582	0.131
	SUVmax	1.14	1.08–1.20	0.000*
	TLG-P	0.98	0.96–1.01	0.109
	TLG-C	1.02	0.99–1.05	0.129
**AC (*n*= 283)**	Sex	0.28	0.02–3.33	0.313
	Smoking	4.70	0.39–55.97	0.221
	SUVmax	1.16	1.07–1.24	0.000*
	TLG-P	0.97	0.93–1.01	0.104
	TLG-C	1.03	0.99–1.07	0.147
**SCC (*n*= 91)**	Sex	0.18	0.01–7.21	0.358
	Smoking	3.40	0.30–38.09	0.320
	Location	1.97	0.57–6.79	0.281
	SUVmax	1.12	1.01–1.24	0.032*
	TLG-P	0.99	0.95–1.02	0.381
	TLG-C	1.01	0.98–1.05	0.407

AC and SCC represent adenocarcinoma and squamous cell carcinoma and *P < 0.05

### PD-L1 correlation with PET/CT and clinicopathological features in pathological subgroups

**Fig. 4:**
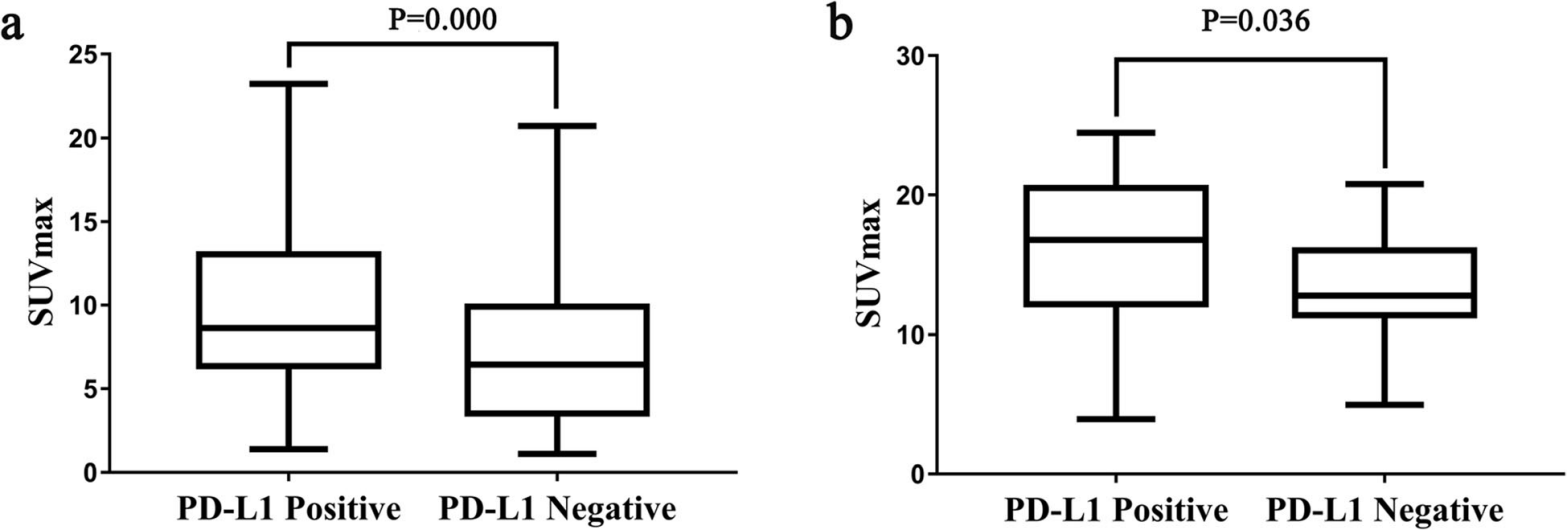
The correlation between SUVmax and PD-L1 expression in AC (**a**) and SCC (**b**), respectively.

The percentage of PD-L1 positive expression was 21.6% (61/283) and 47.3% (43/91) in AC and SCC, respectively. PD-L1 positive cases were significantly higher in SCC than in AC (*P* < 0.05). For both AC and SCC subgroups, SUVmax in PD-L1 positive tumors was significantly higher than that in PD-L1 negative tumors (*P* < 0.05) (Table 1, Fig. 4). And no significant differences in MTV, TLG, and clinicopathological features between PD-L1 positive and negative groups were noted (*P* > 0.05, Table 1).

Multivariate analysis also revealed that SUVmax was an independent predictor of PD-L1 positive expression in both AC and SCC subgroups (Table 2). Based on bootstrapping analysis, the best SUVmax cut-off value was determined to be 7.2 and 16.3 in AC and SCC subgroups, respectively, and the area under the curve was 0.832 (95% CI 0.780–0.884) and 0.848 (95% CI 0.763–0.933), respectively. PD-L1 status of lung AC and SCC could be predicted by FDG PET/CT with sensitivity, specificity, and accuracy of 83.6% vs 69.8%, 60.8% vs 89.6%, and 65.7% vs 71.4%, respectively (Fig. 3b and c).

## Discussion

In recent years, immunotherapy targeting PD-L1 has shown promising results in NSCLC patients. Different TPS values were used in previous studies for evaluating tumor PD-L1 expression, including 1%, 5%, 10%, and 50% [26, 27]. The latest version of the National Comprehensive Cancer Network reported NSCLC patients could benefit from immunotherapy when TPS was over 1% [12]. For example, when TPS ≥ 50%, PD-1/PD-L1 antibody combined with chemotherapy is the best choice for ACC, and PD-1/PD-L1 antibody alone is the best choice for SCC. When TPS during 1% to 49%, PD-1/PD-L1 antibody combined with chemotherapy is prior to PD-1/PD-L1 antibody alone for both ACC and SCC. When TPS < 1%, PD-1/PD-L1 antibody combined with chemotherapy is better than chemotherapy alone for both ACC and SCC [28]. Therefore, we chose TPS ≥ 1% as PD-L1 positive in this study and investigated PD-L1 expression correlation with metabolic parameters of FDG PET/CT and clinicopathological characteristics in NSCLC.

Several reports suggested that the association between the percentages of PD-L1 positive tumors and histological subtypes of NSCLC was found although the criteria of PD-L1 positivity varied in each study. Janzic et al. [26] reported that PD-L1 positive (TPS ≥ 5%) cases were higher in SCC (52%) than in AC (17%). Lin et al. [27] showed that PD-L1 positivity (TPS ≥ 1%) was more frequently observed in SCC (46%) than in AC (27%). Miyazawa et al. [29] compared the percentages of PD-L1 positive NSCLCs using the same criteria as Lin et al. [27], which were confirmed to be more frequent in SCC (44%) or large cell carcinoma (67%) than in AC (21%). In this study, the percentage of tumors with PD-L1 positive expression was 27.8%, 21.6%, and 47.3% in overall NSCLC, AC, and SCC, respectively. PD-L1 positive rate of SCC was significantly higher than that of AC, which was consistent with the previous reports [26, 30]. Moreover, our previous study [22] demonstrated that the positive rate of PD-L1 expression in pulmonary sarcomatoid carcinoma (PSC) was 87.5%, which was higher than that of SCC and AC in this study under the same criteria.

At present, some studies have demonstrated that the predictive value of SUVmax on FDG PET/CT in PD-L1 expression from the primary tumor in patients with lung cancer at the initial diagnosis. For example, Kaira et al. [21] demonstrated that PD-L1 expression level was significantly correlated with SUVmax in lung AC. Zhang et al. [19] reported a significant correlation between PD-L1 expression levels and SUVmax in pulmonary SCC. Takada et al. reported the association between PD-L1 expression and SUVmax in patients with small-sized lung cancer [18] or NSCLC [20], respectively. Our previous study also confirmed that SUVmax could be used to assess PD-L1 expression of PSC [22]. Similar to these previous reports, the results of the present study showed that PD-L1 expression was associated with SUVmax in overall NSCLC, lung AC, and SCC. It may be due to altered metabolism of tumor-infiltrating T lymphocytes resulting from tumor-imposed glucose restriction. Chang et al. [31] showed the glucose consumption by tumors metabolically restricts T cells, leading to their dampened mTOR activity, glycolytic capacity, and IFN-γ production, thereby allowing tumor progression. They also found that blocking PD-L1 directly on tumors dampens glycolysis by inhibiting mTOR activity and decreasing expression of glycolysis enzymes, reflecting a role for PD-L1 in tumor glucose utilization.

However, Jreige et al. [32] reported that no significant correlation was observed between PD-L1 tumor expression and following parameters such as SUV, MTV, and TLG in 49 cases with confirmed NSCLC. SUV represents the FDG activity of the tumor mass. MTV and TLG represent incorporating information on both tumor volume and metabolic factors. These metabolic parameters of PET/CT could reflect the glucose metabolic status of tumor tissues, which is an important feature of tumor biology [23]. Therefore, contrary to the results of Jreige et al. [32], our study found that in addition to SUVmax, TLG-P and TLG-C were also correlated with PD-L1 positivity in NSCLC. With regard to MTV, it represents the metabolic volume of the tumor mass, and different from the calculation of TLG, FDG activity is not included in the measurement of MTV. Our study showed that PD-L1 positivity had no association with the tumor metabolic volume as well as the tumor size. Thus, it demonstrated that the PD-L1-positive expression was mainly associated with FDG activity. Furthermore, when pathologically subgrouped, only SUVmax had the relationship with PD-L1 expression, which further displayed that the relationship between PD-L1 positivity and glucose activity of the tumor mass.

Moreover, this study also demonstrated that PD-L1 expression was significantly correlated with man, smoking ,and central NSCLC, which were also consistent with the previous studies [18, 20, 33]. It suggested that male, smoking-associated and central NSCLC such as lung SCC tended to exhibit PD-L1 positive expression, which confirmed the findings that PD-L1 positive rate of lung SCC was significantly higher than that of lung AC [26, 27, 29]. Furthermore, when the population of this study was pathologically subgrouped, the correlation between PD-L1 expression and these clinicopathological features disappeared, which was also in agreement with others’ reports [9, 34]. It was mainly due to that male, smoking and central tumor were major features of lung SCC, which were eliminated the interrelation with PD-L1 when compared intra-group. Moreover, the relationship between PD-L1 expression and EGFR mutation of NSCLC remains controversial. Several studies suggested that PD-L1 was highly expressed in EGFR wildtype NSCLC [35, 36]; meanwhile, a negative or no correlation between EGFR mutation and PD-L1 expression was also concluded [37–39]. For this study, almost all cases with EGFR mutation were lung ACs, and no obvious difference of PD-L1 expression in EGFR wildtype and EGER mutant tumors was found in overall NSCLC and AC groups, respectively.

Furthermore, although the results of the present study demonstrated that PD-L1 expression of NSCLC was related with SUVmax, TLG, man, smoking, and central location, multivariate analysis displayed that only SUVmax was identified as an independent predictor of PD-L1 positivity in NSCLC, which was in agreement with the previous studies [18, 20]. It may be due to that SUVmax represents a maximally active portion of the tumor, and preferably predicts the tumor aggression. Moreover, similar to the previous reports [19, 21], SUVmax was also an independent and unique predictor of PD-L1 positive expression in AC and SCC subgroups, respectively. Subsequently, the ROC curves and the areas under the curve in this study suggested that SUVmax could predict PD-L1 expression in overall NSCLC, AC, and SCC with high sensitivity, specificity, and accuracy, respectively, which was also in line with the previous reports [18, 20, 21]. Therefore, the SUVmax of FDG PET/CT could be helpful for exploring the existence of immune checkpoints.

Clearly, there were also several limitations in this study. Firstly, based on immunohistochemical requirements of tumor specimens for detecting PD-L1 expression, the patients included in the present study were mainly at stage I–III; therefore, cases at stage IV were quite limited, which was also the limitation of the previous studies. Secondly, due to the invasion of biopsy, the metastatic lesions were usually not biopsied again in routine clinical practice once PD-L1 expression of the primary lesion confirmed. For this study, PD-L1 expression was confirmed by the biopsy of the primary lesion. Thus, the relationship between SUVmax and PD-L1 of metastatic lesions was not analyzed. Although PD-L1 expression may exist a difference in primary and metastatic tumors, Kim et al. [40] demonstrated that the concordance of PD-L1 expression between primary and metastatic pulmonary adenocarcinomas is high when using cutoff values of 1% and 50%. Thirdly, PD-L1 expression was regarded as an indicator of poor prognosis of lung cancer [41–43]. Meanwhile, SUVmax, MTV, and TLG were also reported to have the prognostic values in NSCLC [44, 45]. However, the correlations of PD-L1 expression and PET/CT metabolic parameters with the prognosis were not analyzed, which will be carried out in the future study. Finally, although exploring the existence of PD-L1 could provide strategies to choose immunotherapy for NSCLC, responses to PD-1/PD-L1 antibodies were also noted in NSCLC with low or absent PD-L1 expression. However, due to the small population choosing immunotherapy in this study, the correlations of PET/CT metabolic parameters and immunotherapy were not investigated. Therefore, further large-scale, prospective studies of the role of FDG PET/CT in immunotherapy are warranted.

## Conclusion

PD-L1 expression of NSCLC was significantly related with SUVmax, TLG, man, smoking, and central location. No obvious difference in MTV, age, serum tumor markers, tumor size, TNM stage, and genetic mutation was observed between PD-L1 positive and negative groups. Only SUVmax of FDG PET/CT was an independent predictor of PD-L1 expression, which could help to explore the existence of immune checkpoints.

## Data Availability

The datasets used and/or analyzed during the current study are available from the corresponding author on reasonable request.

## Abbreviations

NSCLC: Non-small cell lung cancer
PD-L1: Programmed cell death ligand-1
FDG: Fluorodeoxyglucose
SUVmax: Maximum standard uptake
MTV: Metabolic tumor volume
TLG: Total lesion glycolysis
AC: Adenocarcinoma
SCC: Squamous cell carcinoma
ROIs: Regions of interest
TPS: Tumor proportion score
EGFR: Epidermal growth factor receptor
KRAS: Kirsten rat sarcoma viral oncogene homolog gene
EML4-ALK: Echinoderm microtubule-associated protein-like 4-anaplastic lymphoma kinase fusion gene
BRAF: V-Raf murine sarcoma viral oncogene homolog B1 proto-oncogene
ROS1: C-Ros oncogene 1
ROC: Receiver operating characteristic
CYFRA211: Cytokeratin 211
CEA: Carcinoembryonic antigen
NSE: Neuron-specific enolase
proGRP: Pro-gastrin-releasing peptide
SCCA: Squamous cell carcinoma antigen
PSC: Pulmonary sarcomatoid carcinoma
